# LncRNA MALAT1 facilitates inflammasome activation via epigenetic suppression of Nrf2 in Parkinson’s disease

**DOI:** 10.1186/s13041-020-00656-8

**Published:** 2020-09-24

**Authors:** Li-Jun Cai, Li Tu, Xiao-Mo Huang, Jia Huang, Nan Qiu, Guang-Hong Xie, Jian-Xiong Liao, Wei Du, Ying-Yue Zhang, Jin-Yong Tian

**Affiliations:** 1 Department of Neurology, the Affiliated Hospital of Guizhou Medical University, Guiyang, 550004 P.R. China; 2 Department of General Medical, the Affiliated Hospital of Guizhou Medical University, Guiyang, 550004 P.R. China; 3 Department of Emergency, Guizhou Provincial People’s Hospital, No.83 Zhongshan East Road, Guiyang, 550002 Guizhou Province P.R. China

**Keywords:** Parkinson’s disease (PD), MALAT1, EZH2, NRF2, Microglia, Inflammasome, ROS

## Abstract

The goal of the present study was to elucidate the mechanism by which long non-coding RNA metastasis-associated lung adenocarcinoma transcript 1 (lncRNA MALAT1) promotes inflammation in Parkinson’s disease (PD). 1-Methyl-4-phenyl-1,2,3,6-tetrahydropyridine (MPTP) was used to induce PD development in C57BL/6 mice, and tyrosine hydroxylase (TH) expression was analysed by immunohistochemical analysis. Western blot and qPCR analyses were conducted to assess the expression of protein and mRNA levels, respectively. Lipopolysaccharide/adenosine triphosphate (LPS/ATP) was used to activate microglia in vitro. Chromatin immunoprecipitation (ChIP), RNA pull-down and RNA immunoprecipitation chip (RIP) assays were performed to investigate the interaction among specific molecules. The 3-(4,5-dimethylthiazol-2-yl)-2,5-diphenyltetrazolium bromide (MTT) assay was used to evaluate cell viability and proliferation. Flow cytometry was performed to analyse cell apoptosis after staining. The dichlorofluorescein diacetate (DCFH-DA) assay was used to measure the generation of reactive oxygen species (ROS) in cells. The results showed that MALAT1 was highly expressed in the brains of MPTP-induced PD model mice and in LPS/ATP-induced microglia cells. Knockdown of MALAT1 inhibited elevated nuclear factor (erythroid-derived 2)-like-2 factor (NRF2) expression, thereby inhibiting inflammasome activation and ROS production. MALAT1 was shown to promote neuroinflammation by recruiting enhancer of zeste homologue 2 (EZH2) to the promoter of NRF2, suppressing Nrf2 expression. In summary, MALAT1 epigenetically inhibits NRF2, thereby inducing inflammasome activation and reactive oxygen species (ROS) production in PD mouse and microglial cell models.

## Introduction

Parkinson’s disease (PD) is an age-related degenerative disorder of the nervous system that affects the movement abilities of adults [1]. Every year, approximately 8–18 of every 100,000 individuals are newly diagnosed with PD [2]. The selective loss of dopaminergic neurons in the substantia nigra (SN) pars compacta of PD patients has been demonstrated. Furthermore, the development of intracellular α-synuclein (α-syn) aggregates is an epigenetic characteristic of PD development and have been shown to be associated with movement coordination impairment and cognitive deficits [3]. In addition, the inflammasome expression observed in the brains of PD patients indicates that inflammation also contributes to the acceleration of PD development [4]. The inflammasome has been reported to be involved in the pathogenesis of PD [5]. For instance, the nucleotide-binding domain (NOD)-like receptor protein 3 (NLRP3) inflammasome, including NLRP3, caspase-1 and cytokines of the interleukin-1 (IL-1) family, was shown to be activated in LPS- and 6-hydroxydopamine hydrobromide (6-OHDA)-induced PD rats [6]. In the central nervous system, the NLRP3 inflammasome signalling pathway plays a crucial role in neuroinflammatory processes, such as neurodegenerative diseases. Sarkar et al. recently demonstrated that amplification of NLRP3 inflammasome activation by mitochondrial impairment in microglia promotes the development of PD [7]. At present, there is no neuroprotective therapy to treat or halt the progress of PD except for strategies to relieve the associated symptoms [8]. Therefore, there is an urgent need to understand the pathogenic mechanism of PD at the molecular level and identify potential targets to develop novel therapeutic strategies for this disease.

Non-coding RNAs (ncRNAs), including long non-coding RNAs (lncRNAs) and microRNAs (miRNAs), have important roles in many cellular processes and pathological diseases [9, 10]. LncRNAs, a class of ncRNAs greater than 200 nucleotides with no protein coding ability, have been demonstrated to be highly expressed in the brain and participate in numerous neurobiological functions, as well as in tumour tissues [11–13]. In PD patients and PD mouse models, many lncRNAs have been shown to be upregulated in the brain, indicating that they may be involved in many neurobiological progress and neurodegenerative disorders [14]. For instance, 756 lncRNAs were reported to be abnormally expressed in transgenic mice together with **α**-synuclein compared to that observed in the control groups [15]. Chen et al. recently reported that lncRNA small nucleolar RNA host gene 1 (lncRNA SNHG1) is upregulated in PD, which promotes **α**-synuclein aggregation and toxicity by targeting miR-15b-5p [16]. Metastasis-associated lung adenocarcinoma transcript 1 (MALAT1), also known as nuclear-enriched abundant transcript 2 (NEAT2), is a highly conserved lncRNA that is typically upregulated in various tumour tissues [17] and disordered neurons [18]. Recently, MALAT1 was demonstrated to be involved in dendritic and synapse development [19] and plays an important regulating role in MPTP-induced PD [20]. However, the detailed mechanism by which MALAT1 regulates PD development has not been fully elucidated.

Nuclear factor (erythroid-derived 2)-like-2 factor (Nrf2) has recently attracted attention as a redox-sensitive transcription factor [21] that is activated under highly oxidizing conditions to regulate target cytoprotective genes, such as haem oxygenase-1 (HO-1, [22]). Low expression of Nrf2 promotes the inflammation process [23]. Nrf2 was demonstrated to contribute to the anti-inflammatory process by recruiting inflammatory cells and regulating gene expression of antioxidant response elements [23]. For instance, Choi et al. reported that the Nrf2 activation by vinyl sulfone derivatives could be used for PD therapy [24]. Okorji et al. observed that the antimalarial drug artemether can induce the expression of Nrf2 to regulate ROS expression levels in BV2 microglia [21]. In addition, Rojo et al. demonstrated that Nrf2 can regulate microglial activation to promote neuroinflammation in PD [25]. However, the interaction between MALAT1 and Nrf2 in the development of neurodegeneration disease remains unclear.

In the present study, we investigated the role of MALAT1 in the development of PD and assessed the interaction between MALAT1 and Nrf2 in PD. The results showed that after the induction of LPS/ATP in BV2 microglia, the expression of MALAT1 was significantly increased as well as in the PD models induced by MPTP. The mechanistic investigation demonstrated that MALAT1 inhibits Nrf2 by regulating EZH2-mediated epigenetic repression, thereby leading to increased ROS levels and inflammasome activation.

## Materials and methods

### Animals

Male C57BL/6 mice (6–8 weeks of age) were obtained from the Hunan SJAC Laboratory Animal (Changsha, China). All animal experiments were approved by and conformed to the guidelines of Guizhou Provincial People’s Hospital. The C57BL/6 mice were housed under a 12 h dark and light cycle with free access to water and food and randomly divided into PD and control groups. To establish a PD model, mice were intraperitoneally injected with the neurotoxin 1-methyl-4-phenyl-1,2,3,6-tetrahydropyridine-hydrochloride (MPTP-hydrochloride, 20 mg/kg) (Sigma-Aldrich, St. Louis, MO, USA) 3 times each day, while mice in the control group were injected with sterile saline solution (0.9%). Ventral midbrains of all mice were collected after sacrifice and stored at − 80 °C for further usage. For in vivo knockdown of MALAT1, siNC or siMALAT1 lentiviruses (2 × 10^8^ viral genome/μl, GenePharma, Shanghai, China) were injected into the lateral ventricle of C57BL/6 J mice (0.3 mm after bregma, 1.0 mm after sagittal line, and at a depth of 2.2 mm from the skull surface). Two weeks after injection, the mice was subjected treated to generate the PD model as described above.

### Rotarod test

The rotarod test, which measures coordinate motor skill, was used to evaluate the behaviour of the PD and control mice. An accelerated protocol of the rotarod test was performed as previously described [26]. The test was repeated five times a day (averaged as the mean in one day) for each animal over three consecutive days, and the sum of the mean values from the three days was presented as the movement time.

### Immunohistochemistry

The substantia nigra regions of the brains of PD and control mice were collected and fixed in 4% paraformaldehyde. Following serial coronal sectioning (30-μm sections), the brain tissues were blocked with 5% goat or horse serum/PBS plus 0.2% Triton X-100. Then, the sections were incubated with an anti-TH antibody (Novus Biologicals) or an anti-Iba-1 antibody (Wako) before being incubated with a biotin-conjugated anti-rabbit antibody (Vector Labs). After 2 h, the sections were washed with PBS three times, incubated with ABC reagents (Vector Labs) and then developed using Sigma Fast 3,3′-Diaminobenzidine (DAB) Peroxidase Substrate (Sigma-Aldrich, St. Louis, MO, USA). Nissl (0.99% thionin) was used to counterstain the sections.

### Cell culture and treatment

The BV2 mouse microglia cell line and the human neuro cell line N2a were purchased from the Chinese Academy of Sciences Collection Committee Cell Bank (Shanghai, China). BV2 cells were cultured and passaged in RPMI 1640 supplemented with 10% foetal bovine serum (Sigma-Aldrich, St. Louis, MO, USA), 100 U/mL penicillin (Sigma-Aldrich, St. Louis, MO, USA) and 100 μg/mL streptomycin under an atmosphere with 5% CO_2_ at 37 °C. Subsequently, BV2 cells were seeded in a 96-well plate at a density of 2 × 10^5^/mL and then treated with 10 ng/mL LPS (Sigma-Aldrich, St. Louis, MO, USA) for 4 h. Then, the cells were treated with 5 mM ATP (Sigma-Aldrich, St. Louis, MO, USA) for another 30 min. N2a cells were cultured in DMEM supplemented with 10% foetal bovine serum (Sigma-Aldrich, St. Louis, MO, USA), 100 U/mL penicillin (Sigma-Aldrich, St. Louis, MO, USA) and 100 μg/mL streptomycin in an incubator under an atmosphere with 5% CO_2_ at 37 °C.

### Cell transfection

Cells were transfected with sh-NC, sh-MALAT1, and a MALAT1 vector, which were purchased from GenePharma (Shanghai, China), to modulate the expression of MALAT1 in the absence or presence of LPS. The sequence of sh-MALAT1 was 5′-AAGGATGTCAGCGCACTAAAT-3′. Lipofectamine 3000 (Invitrogen, USA) was used for cell transfection according to the manufacturer’s protocols. ML385 and GSK-126 were used to inhibit Nrf2 or EZH2, respectively. The effect of MALAT1-transfected microglia on neurons was investigated using a Transwell co-culture system. Briefly, an N2a neuronal cell was added to the lower chamber of the 96-well plate, and the BV2 cells with different treatments were cultured on the Transwell inserts (pore size 0.4 μm; Corning, USA) in the 96-well plate.

### Cell viability assay

The MTT assay (Roche Molecular Bio- chemicals, Rotkreuz, Switzerland) was used to evaluate the proliferation of BV2 cells with different treatments. Briefly, BV2 cells seeded at a density of 3 × 10^3^ cells/well in 96-well plates were treated with different concentrations of LPS (0, 100, 500, and 1000 ng/mL) or for different treatment times (1, 2, 4, 6, 8 h). The cells without any treatment were used as a control. After the addition of MTT reagent and a 4-h incubation in an incubator under an atmosphere with CO_2_, the medium was removed. Then, 200 μL of DMSO was added into each well, and the absorbance was read at 450 nm on a microplate reader.

### Elisa

Briefly, the cultured BV2 microglia with different treatments were centrifuged to collect the culture supernatants. Then, the concentrations of TNF-α, IL-1β and IL-18 in the supernatants were determined using commercially available ELISA kits (Boster, Wuhan, China) according to the manufacturer’s instructions. The quantitative measurements were recorded using a plate reader at a wavelength of 450 nm.

### Apoptosis analysis by flow cytometry

To analyse the apoptosis rate of the cells after different treatments, the cells were stained using an Annexin V-FITC/PI Apoptosis Detection kit (BD Biosciences) according to the manufacturer’s instructions. Then, the cells were analysed by flow cytometry, and the apoptosis rate of the cells was quantified using FlowJo.

### Intracellular ROS measurement

The ROS levels in the BV2 and N2a cells were measured using a 2,7-dichlorofluorescein diacetate (DCFH-DA) Cellular ROS Assay kit (Abcam). The cells seeded in the plates were incubated with 10 μM DCFH-DA for 30 min at 37 °C. Then, the cells were washed with fresh medium to remove excess DCFH-DA followed by different treatments and stimulation by LPS/ATP. Subsequently, the cells were imaged using a fluorescent microscope with excitation at 488 nm.

### Quantitative reverse transcription polymerase chain reaction (RT-qPCR)

Total RNA was isolated from the mouse brain tissues and BV2 cells using an RNA-Pure kit (Tiangen, Beijing, China) according to the manufacturer’s instructions. RNA was reverse transcribed into complementary DNA (cDNA) with a SuperScript cDNA Synthesis kit (Invitrogen) followed by an analysis with GoTaq qPCR Master Mix (Promega) according to the manufacturer’s protocol using SYBR Green (Invitrogen, Carlsbad, CA) and was performed on a Step One Realtime PCR System (Applied Biosystems, Foster City, CA). The specific primers for MALAT1, TNF-α, IL-1β, IL-18, HO-1, NQO-1, and SOD2 were designed and synthesized by Sangon Biotech (Shanghai). GAPDH was used as control.

### Western blot

To analyse the expression level of proteins in the brain tissues and cells, NP-40 buffer (150 mM sodium chloride, 1.0% NP-40, 0.5% sodium deoxycholate, 0.1% sodium dodecyl sulfate, and 50 mM Tris, pH 8.0) supplemented with 1 mM phenylmethylsulfonyl fluoride was used to obtain protein samples. Then, the protein lysates were separated on sodium dodecyl sulfate-polyacrylamide gels. The proteins were then transferred to polyvinylidene difluoride membranes, which were blocked with 2% bovine serum albumin (BSA). The membranes were then incubated with the primary antibodies, including antibodies against NLRP3 (#15101), ASC (#67824), cleaved caspase 1 (#89332), Nrf2 (#12721 T), HO-1 (#86806), EZH2 (#5246), H3K27me3 (#9733) and β-actin were purchased from Cell Signaling Technology (MA, USA), followed by an incubation with the appropriate peroxidase-conjugated secondary antibodies (ZSGB-Bio, Beijing, China). An ECL detection kit (Thermo Fisher Scientific, Waltham, MA) was used to image and quantify the immunoreactive bands.

### RNA pull-down experiment

In the present study, the interaction between MALAT1 and EZH2 was assessed using a BersinBioTM RNA pull-down kit (ThermoFisher Scientific, USA). Biotin-labelled MALAT1 and its antisense RNA were obtained using RNA biotin labelling mix, and the RNA pull-down assay was conducted as previously described [27]. Human antigen R (HuR) and ARE-mRNAs (AR) were used as positive controls.

### RNA-binding protein immunoprecipitation (RIP) assay

For the RIP experiments, an EZ-Magna RIP™ RNA-Binding Protein Immunoprecipitation kit (Millipore, USA) and a mouse anti-EZH2 antibody (Abcam) was used according to the manufacturer’s instructions. Briefly, BV2 cells were collected and lysed with the buffer provided in the kit, and the anti-EZH2 antibody or control IgG (negative control) was used to perform the immunoprecipitation. After purification with RNAiso Plus (Takara, Japan), RT-qPCR analysis was performed to evaluate the RNA levels [28]. RIP enrichment represented the fold change of lncRNA immunoprecipitated by the antibody against EZH2 compared to that immunoprecipitated by IgG.

### Chromatin immunoprecipitation (ChIP)

ChIP was performed using a kit (Millipore, 17–295) according to the manufacturer’s instructions. BV2 cells were used to assess the binding of EZH2 and H3K27me3 with the Nrf2 promotor as described in a previous publication [4]. Briefly, the BV2 cells were digested in digestion buffer (50 mM Tris-Cl (pH 7.6), 1 mM CaCl_2_, 0.2% Triton X-100, 5 mM butyrate, 1× protease inhibitor cocktail, and 0.5 mM PMSF). Subsequently, 0.3 U of micrococcal nuclease (MNase; Sigma-Aldrich, St. Louis, MO) was added to the solution, which was then incubated for 5 min at 37 °C. After inactivation of the reaction by incubation with 50 mM EDTA and RIPA buffer for 16 h, approximately 3 μg of primary antibody against EZH2 (#5246) and H3K27me3 coupled to Dynabeads Protein A beads (#9733, Invitrogen) were incubated with the solution for 16 h at 4 °C. Normal rabbit IgG (Santa Cruz sc-2025) was used as a control. The DNA was extracted and treated according to the instructions provided with the ChIP Kit and was used for PCR analysis. The immunoprecipitated DNA was for PCR analysis, and the relative enrichment levels were determined as the fold changes compared with the IgG control.

### Statistical analysis

All quantitative experiments were performed at least three times, and the results are presented as the means ± SD. Unpaired two-tailed Student’s t-test was used to compare the difference between two groups. Comparisons among multiple groups were analysed by one-way ANOVA. Differences were considered significant at *P* < 0.05 (*p < 0.05, ** *p* < 0.01, and *** *p* < 0.001).

## Results

### Highly expressed MALAT1 and suppressed Nrf2 are coupled with inflammasome activation in the brain of PD mice

**Fig. 1:**
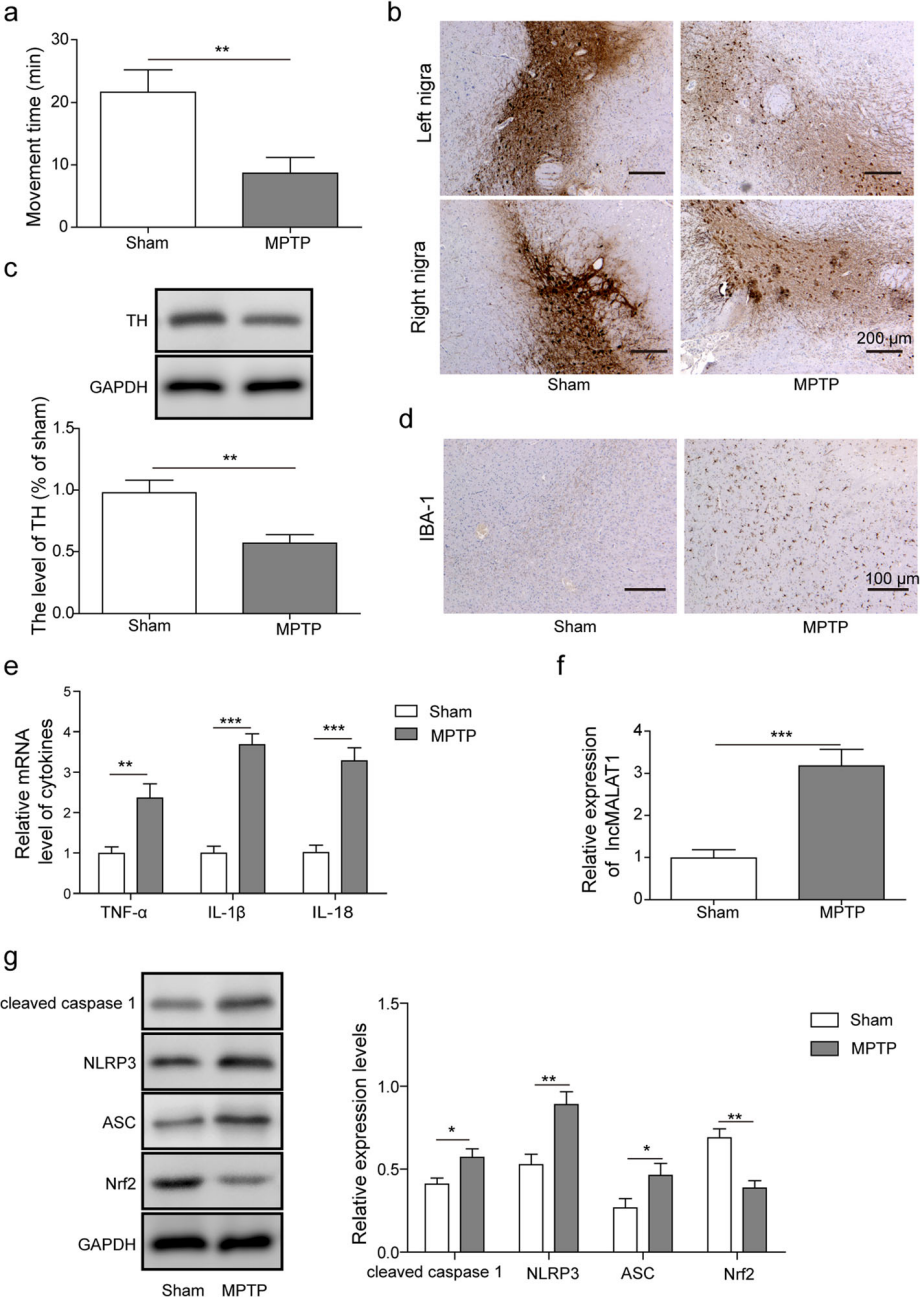
MPTP-induced PD mice show activation of inflammasome (**a**) Rotarod assay of the mice intraperitoneally treated with MPTP. Movement time: latency of an animal dropping off from rotating rod (**b**) The number of TH+ positive neurons was decreased in PD mouse brains by immunohistochemical analysis. **c** The expression level of TH in the brains of PD mice was analysed by western blot analysis. **d** The immunohistochemical analysis of IBA-1 in the microglial cells in the PD mice. **e** The mRNA levels of TNF-α, IL-1β and IL-18 were measured in PD mice qPCR. **f** The mRNA level of MALAT1 was determined in PD mice by qPCR. **g** Western blot analysis of cleaved caspase 1, NLRP3, ASC and Nrf2 levels in PD mice. The data in the graph are presented as the means ±SD as the relative levels from three replications. *n* = 6 for control group, *n* = 8 for PD group. *p < 0.05, **p < 0.01

Mouse PD models (*n* = 8) were established by intraperitoneally injecting mice with MPTP, and mice treated with 0.9% sterile saline solution were used as the control group (*n* = 6). The motor ability of the mice was evaluated using the rotarod test, and the results demonstrated that there was a significant alteration in motor function between the control and MPTP-induced mice (Fig. 1a). The average times the mice spent on the rod were approximately 10 and 23 min for the PD and control mice, respectively. The immunohistochemical and western blot analysis results indicated that TH expression in the brain tissues of the MPTP-induced mice was significantly decreased compared with that observed in the control mice (Fig. 1b and c). Activation of microglia in the brains of PD mice was also significantly increased compared with that observed in the control group by assessing the amount of IBA-1 through IHC (Fig. 1d). RT-qPCR results showed that the levels of pro-inflammatory cytokines, including TNF-α, IL-1β and IL-18, in the MPTP-induced mice brain tissues were also significantly increased compared with that observed in the control group, indicating the activation of neuroinflammation in the MPTP-induced mice (Fig. 1e). Then, the expression of MALAT1 in the MPTP-induced PD mice and control mice was evaluated by RT-qPCR. The results indicated a substantial enhancement of MALAT1 mRNA expression in the MPTP-induced PD mouse brains tissue compared with that observed in the control mice (Fig. 1f). Through western blot analysis, the protein levels of cleaved caspase 1, NLRP3, ASC, and Nrf2 were assessed in the MPTP-induced PD mice and control mice, proteins that are associated with NLRP3-mediated inflammasome activation. The results demonstrated that the expression of cleaved caspase 1, NLRP3, and ASC was elevated in PD mouse brains, whereas that of the antioxidant transcription factor Nrf2 was attenuated in PD mouse brains (Fig. 1g). Taken together, these data suggested that in PD mice, MALAT1 expression was upregulated along with inflammasome activation.

### Knockdown of MALAT1 attenuates inflammasome activation induced by LPS/ATP treatment in BV2 cells

**Fig. 2:**
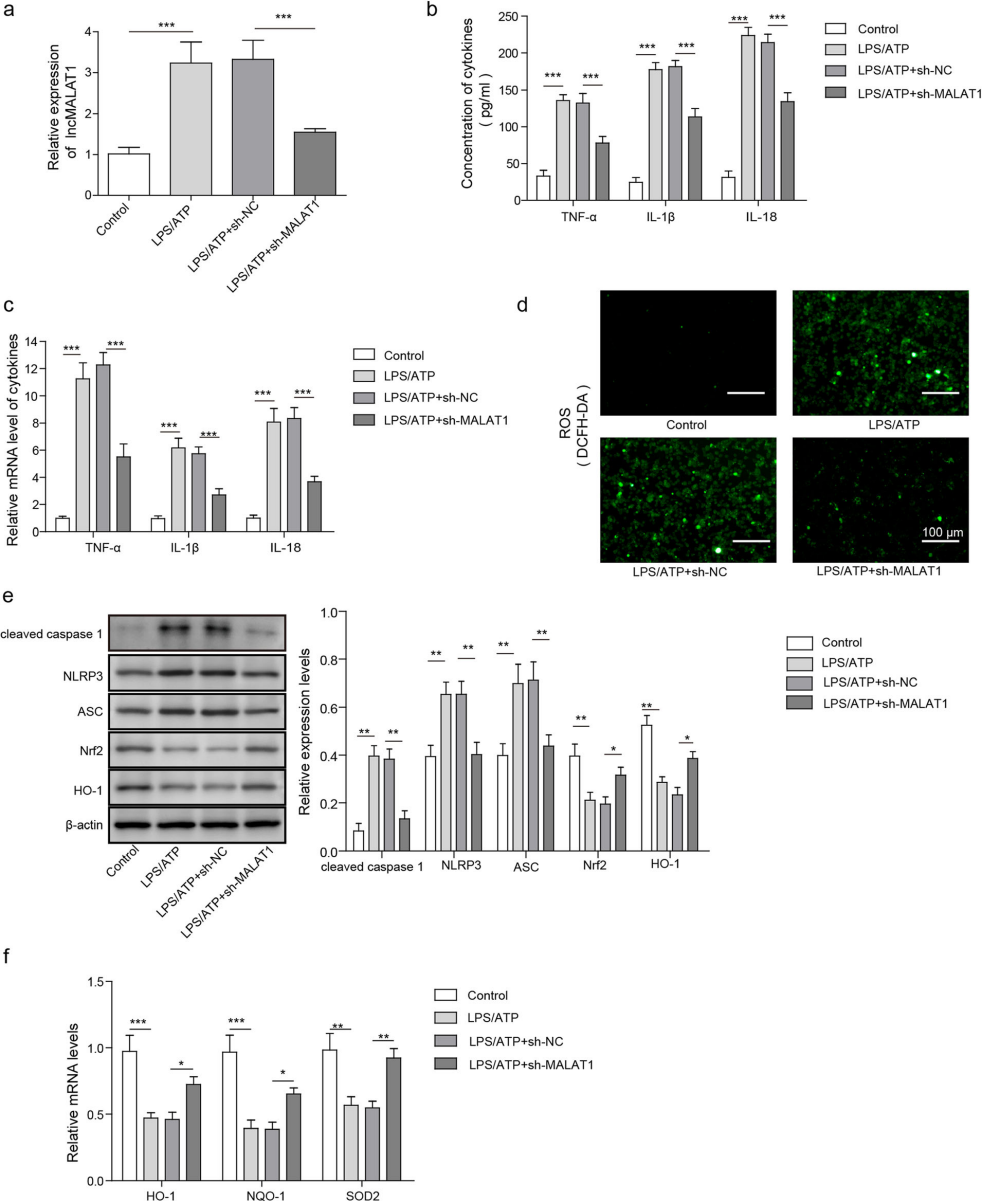
Knockdown of MALAT1 attenuates neuroinflammation caused by LPS/ATP treatment in BV2 cells. After the BV2 cells were treated with LPS/ATP, LPS/ATP + sh-NC, and LPS/ATP + sh-MALAT1, **a** the expression of MALAT1 was analysed by qPCR. **b** The analysis of the cytokines TNF-α, IL-1β and IL-18 through ELISA for the different groups. **c** The mRNA levels of TNF-α, IL-1β and IL-18 were measured by qPCR in different groups. **d** The ROS levels in different groups were determined by fluorescence imaging of DCFH-DA. **e** The western blot analysis of cleaved caspase 1, NLRP3, ASC, Nrf2 and HO-1 levels in different groups. **f** The mRNA levels of HO-1, NQO-1, and SOD2 in different groups were measured by qPCR. The data in the graph are presented as the means ± SD as the relative levels from three replications. *p < 0.05, **p < 0.01, ***p < 0.001

Next, we investigated the effect of MALAT1 knockdown on inflammasome activation in BV2 cells. The BV2 cells were treated with sh-NC or sh-MALAT1 (shRNA targeting MALAT1) 48 h before LPS/ATP treatment, which was applied to induce inflammasome activation. As shown in Fig. 2a, the expression of MALAT1 was significantly increased when the BV2 cells were treated with LPS/TAP compared with that observed in the control group. Transfection with sh-MALAT1 partially reversed the enhanced level of MALAT1 induced by LPS/ATP. The ELISA and RT-qPCR results also showed that the expression of assayed cytokines, including TNF-α, IL-1β and IL-18 were all significantly increased in the BV2 cells treated with LPS/ATP compared with that observed in the control group (Fig. 2b&c). However, knockdown of MALAT1 by sh-MALAT1 in the LPS/ATP-treated BV2 cells reversed the increased expression of these cytokines (Fig. 2b&c). We also assessed the generation of ROS in BV2 cells after different treatments. The results presented in Fig. 2d demonstrated that BV2 cells activated by LPS/ATP showed significantly enhanced ROS levels (brighter fluorescence intensity) compared with that observed in the control group. However, silencing MALAT1 significantly inhibited the fluorescence intensity, indicating lower ROS levels in these cells. The protein expression levels of cleaved caspase 1, NLRP3, ASC, Nrf2, and HO-1 were quantified by western blot in BV2 cells with different treatments. The results showed that LPS/ATP treatment significantly enhanced the expression of cleaved caspase 1, NLRP3, and ASC, while that of Nrf2 and HO-1 (Fig. 2e) was decreased. In contrast, silencing MALAT1 in the LPS/ATP-treated BV2 cells significantly reversed these trends. Moreover. In with respect to mRNA expression levels, we found that LPS/ATP treatment of BV2 cells significantly decreased the expression of Nrf2 downstream antioxidant genes, including HO-1, NQO-1, and SOD2, while silencing MALAT1 by sh-MALAT1 treatment partially restored their expression in BV2 cells (Fig. 2f). Taken together, these results demonstrated that MALAT1 knockdown attenuated inflammasome activation and recovered the Nrf2-mediated antioxidative capability in LPS/ATP-induced BV2 cells.

### MALAT1 binds EZH2 to promote its epigenetic inhibition of Nrf2 in BV2 cells

**Fig. 3:**
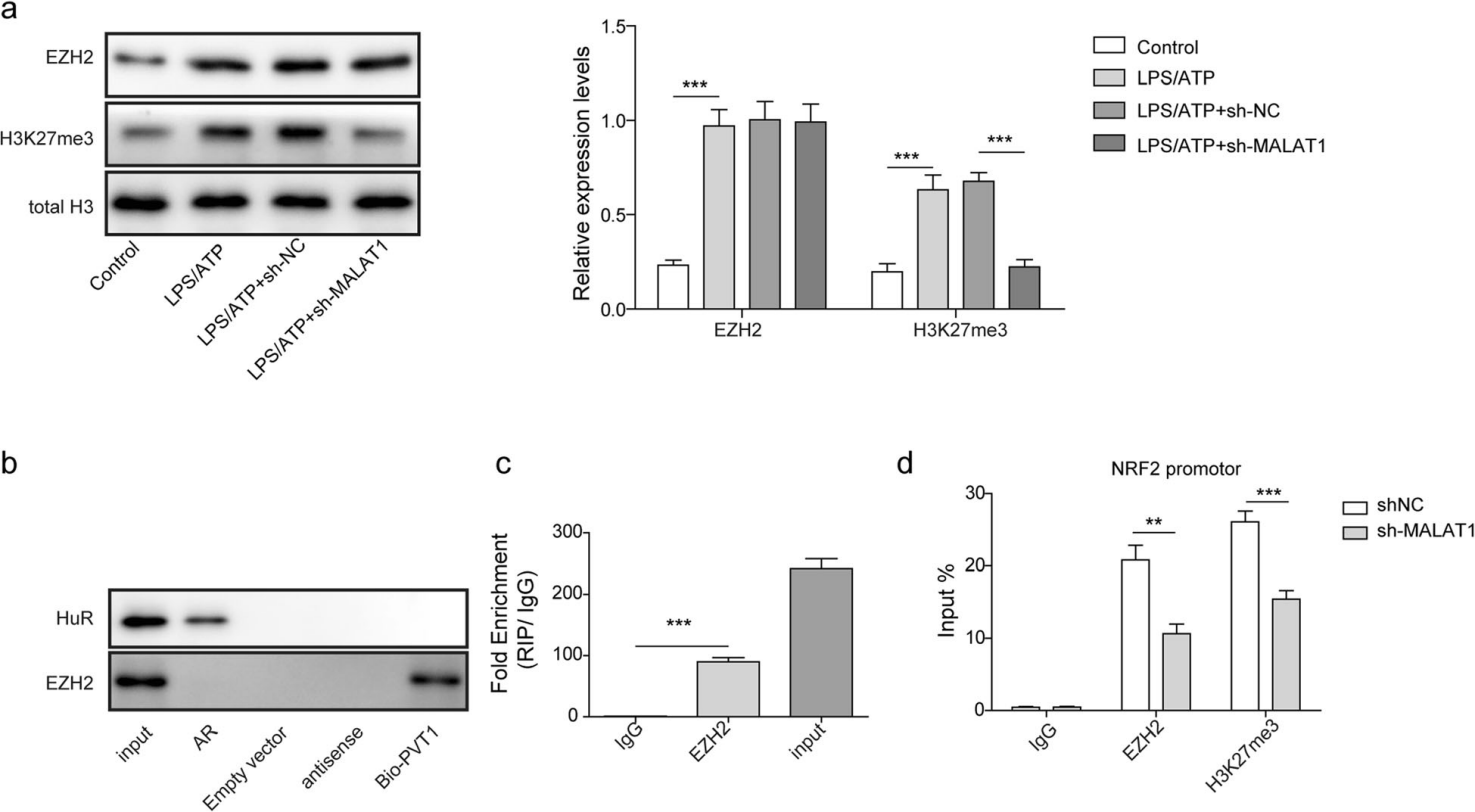
MALAT1 binds EZH2 to recruit h3k27me3 and regulate the expression of Nrf2. After the BV2 cells were treated with LPS/ATP, LPS/ATP + sh-NC, and LPS/ATP + sh-MALAT1 (**a**) The expression of EZH2 and H3K27me3 was analysed by western blot. **b** The combination of MALAT1 and EZH2 analysed by RNA pull-down assay and EZH2 protein levels were detected by western blotting. Human antigen R (HuR) and ARE-mRNAs (AR) were used as negative control (**c**) RNA immunoprecipitation (RIP) experiments was performed in BV2 cells with an antibody EZH2, and the MALAT1 expression was detected by qPCR. **d** ChIP assay was used to investigate the NRF2 promoter expression after treating BV2 cells with shNC or shMALAT1. The data in the graph are presented as the means ± SD as the relative levels from three replications. *p < 0.05, **p < 0.01, ***p < 0.001

Subsequently, the mechanism by which MALAT1 regulates NRF2 and downstream pathways was investigated. Recently, Hirata et al. showed that MALAT1 can promote renal cell carcinoma by regulating EZH2 and the downstream pathway. In the present study, we first assessed the expression of EZH2 and H3K27me3 in different BV2 cell treatment groups by western blot analysis. As shown in Fig. 3a, after LPS/ATP treatment, the levels of EZH2 and H3K27me3 expression were significantly enhanced compared with those observed in the control group. Silencing MALAT1 in the LPS/ATP-treated BV2 cells did not alter the level of EZH2 expression, whereas decreased H3K27me3 expression was observed in the LPS/ATP-treated BV2 cells. To validate the interaction between MALAT1 and EZH2, RNA pull-down assays were performed. The results showed that EZH2 bands could be detected in the Bio-MALAT1 group, while no bands were observed in the empty vector or antisense groups (Fig. 3b), indicating binding between MALAT1 and EZH2. In addition, RNA immunoprecipitation (RIP) results also showed that the group treated with the EZH2 antibody exhibited significantly enhanced RIP enrichment of MALAT1 compared with that observed in the group treated with IgG (Fig. 3c). Furthermore, we investigated the impact of MALAT1 knockdown on the recruitment of EZH2 and H3K27me3 in the promotor region of Nrf2. As shown in Fig. 3d, the ChIP assay results demonstrated that the accumulation of H3K27me3 and EZH2 in the Nrf2 promotor region was significantly decreased upon MALAT1 knockdown. These findings indicate that MALAT1 regulates the expression of Nrf2 through EZH2-mediated epigenetic suppression.

### Silencing MALAT1 inhibits inflammasome activation and ROS generation through an Nrf2-dependent mechanism

**Fig. 4:**
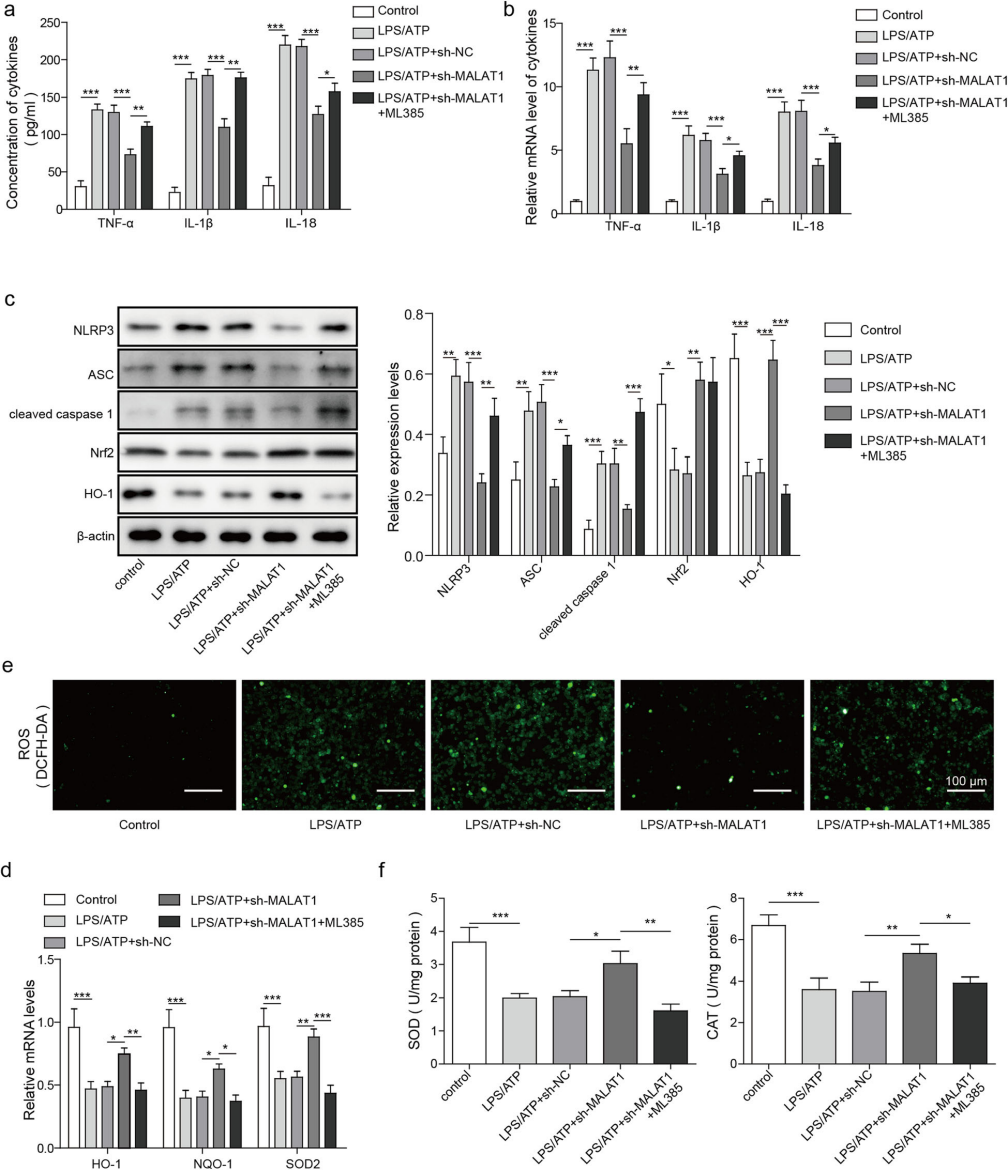
MALAT1 activates the inflammasome through regulation of NRF2. BV2 cells were treated with LPS/ATP, LPS/ATP + sh-NC, LPS/ATP + sh-MALAT1, and LPS/ATP + sh-MALAT1 + ML385. **a** The analysis of the cytokines TNF-α, IL-1β and IL-18 through ELISA. **b** The mRNA levels of TNF-α, IL-1β and IL-18 in BV2 cells were analysed by qPCR. **c** The expression of inflammasome-related proteins, including NLRP3, ASC, cleaved caspase 1, NRF2, and HO-1 in BV2 cells were assessed by western blot analysis. **d** The mRNA levels of HO-1, NQO-1, and SOD2 in the BV2 cells were measured by qPCR. **e** The ROS levels in BV2 cells were assessed using DCFH-DA. **f** Measurements of antioxidants, including SOD and CAT activities, in BV2 cells by qPCR. The data in the graph are presented as the means ±SD as the relative levels from three replications. *p < 0.05, **p < 0.01

To investigate whether the function of MALAT1 on inflammasome activation and ROS generation is associated with the inhibition of Nrf2, MALAT1 expression was silenced in BV2 cells by treatment with sh-MALAT1 alone or together with ML385, an effective inhibitor for Nrf2 Silencing MALAT1 in the LPS/ATP-treated BV2 cells attenuated neuroinflammation, as indicated by the decreased cytokine expression, the levels of which were analysed by ELISA and RT-qPCR (Fig. 4a-b). Interestingly, co-treatment of ML385 with sh-MALAT1 restored the expression of the assayed cytokines. Furthermore, the expression of inflammasome-related proteins including NLRP3, ASC, cleaved caspase 1, NRF2, and HO-1 were in the different treatment groups were assessed by western blot analysis. Knockdown of MALAT1 in BV2 cells after LPS/ATP induction significantly decreased the expression of NLRP3, ASC, and cleaved caspase 1, while that of Nrf2 and HO-1 was increased. In contrast, Nrf2 inhibition by ML385 abrogated the effect of MALAT1 knockdown (Fig. 4c). Additionally, the expression of antioxidative genes, including HO-1, NGO-1, and SOD2 were analysed by RT-qPCR (Fig. 4d). Silencing MALAT1 restored the LPS/ATP-mediated decrease in antioxidative gene expression, while simultaneous treatment ML385 abrogated the effect of MALAT1 knockdown (Fig. 4d). To assess the effect of MALAT1 on neuroinflammation, ROS, SOD, and CAT activities were analysed (Fig. 4e-f). The fluorescence intensity of DCFH-DA in the LPS/ATP-induced BV2 cells were significantly higher than that observed in the control groups, indicating enhanced ROS levels. However, although silencing MALAT1 significantly decreased ROS levels, which were restored by the inhibition of Nrf2 through ML385 treatment (Fig. 4e), SOD and CAT activities were increased when the MALAT1 was knocked out, and treatment of ML385 attenuated this effect (Fig. 4f). Taken together, these results indicate that the Nrf2 signalling pathway may be partly involved in MALAT1-mediated inflammasome activation.

### MALAT1 activates the inflammasome and ROS through EZH2

**Fig. 5:**
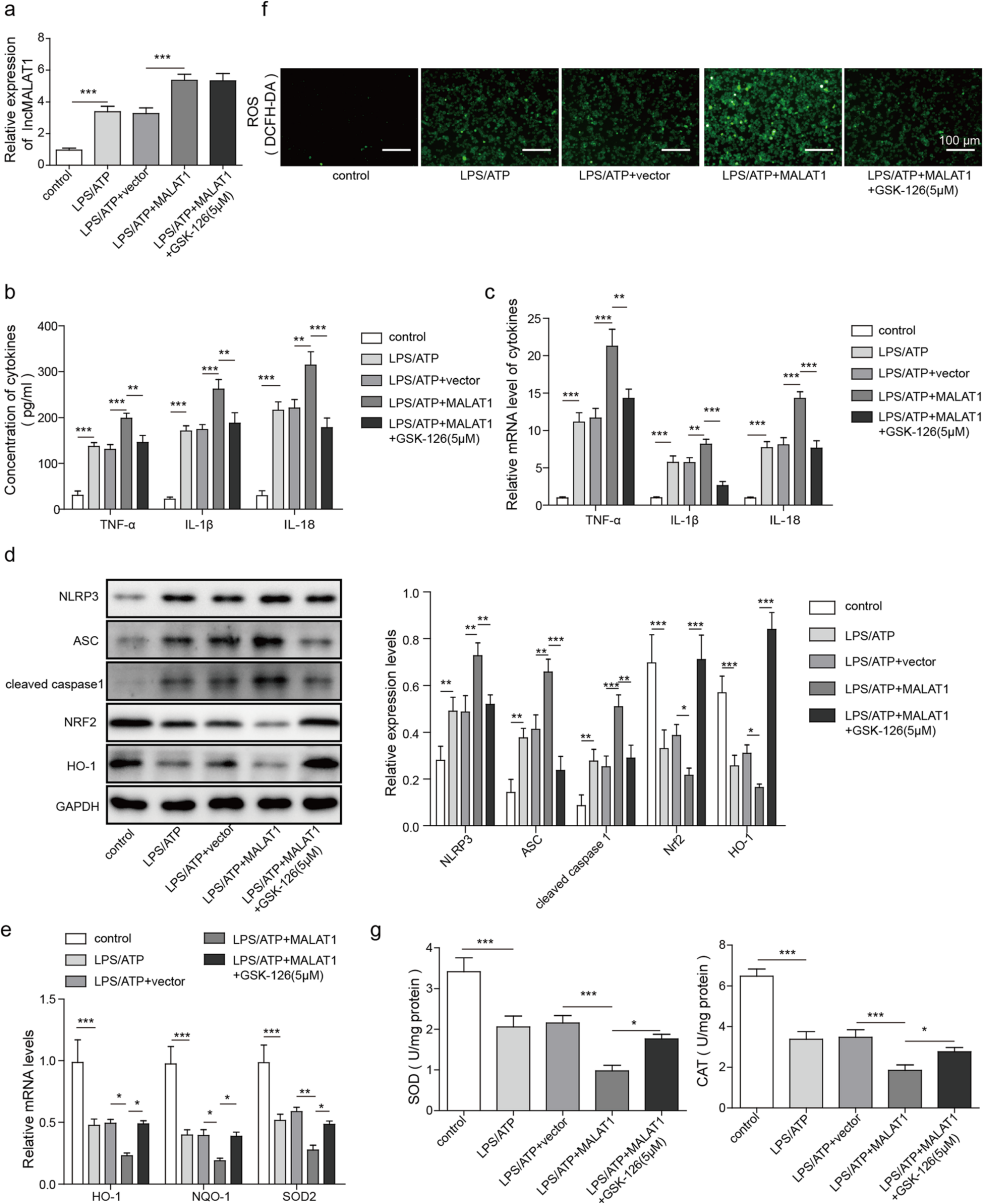
MALAT1 activates the inflammasome through regulation of EZH2. BV2 cells were treated with LPS/ATP, LPS/ATP + sh-NC, LPS/ATP + sh-MALAT1, and LPS/ATP + sh-MALAT1 + GSK-126. **a** The expression of MALAT1 after the BV2 cells were analysed by qPCR. **b** The analysis of the cytokines TNF-α, IL-1β and IL-18 through ELISA. **c** The mRNA levels of TNF-α, IL-1β and IL-18 were determined by qPCR. **d** The expression of inflammasome-related proteins, including NLRP3, ASC, cleaved caspase 1, NRF2, and HO-1 in BV2 cells were assessed by western blot analysis. **e** The mRNA levels of HO-1, NQO-1, and SOD2 in the BV2 cells as assessed by qPCR. **f** The ROS levels in BV2 cells were assessed using DCFH-DA. **f** Measurements of antioxidants, including SOD and CAT activities in BV2 cells. The data in the graph are presented as the means ±SD as the relative levels from three replications. *p < 0.05, **p < 0.01

Subsequently, we investigated the effect of EZH2 in MALAT1-mediated inflammasome activation. To this end, the effects of MALAT1 overexpression or treatment with the EZH2 inhibitor GSK-126 on LPS/ATP-treated BV2 cells were assessed. The results showed that LPS/ATP treatment and MALAT1 overexpression both elevated MALAT1 expression, but GSK-126 had no effect on MALAT1 expression (Fig. 5a). MALAT1 overexpression promoted the neuroinflammation induced by LPS/ATP treatment, as indicated by the increased expression of cytokines, including TNF-α, IL-1β and IL-18 (Fig. 5 c-d). However, inhibition of EZH2 by GSK-126 reversed the effect of MALAT1 overexpression on the levels of these cytokine in the LPS/ATP-treated BV2 cells (Fig. 5b-c). Similarly, the levels of inflammasome-related proteins, such as NLRP3, ASC and cleaved caspase 1, increased with the MALAT1 overexpression but were restored to lower levels when the GSK-126 was introduced (Fig. 5d). MALAT1 overexpression also decreased the expression of antioxidant genes, including HO-1, NQO-1, and SOD2 (Fig. 5e), compared with that observed in BV2 cells only treated with LPS/ATP or/and empty vector. However, treatment with GSK-126 reversed the effect of MALAT1 overexpression in the LPS/ATP-treated BV2 cells. The observed ROS levels (Fig. 5f) and SOD and CAT activities (Fig. 5g) indicated that MALAT1 overexpression inhibited the antioxidative capabilities of cells that were comprised by GSK-126 incubation. Therefore, these results show that MALAT1 may promote ROS levels and inflammasome activation through EZH2.

### Silencing MALAT1 protects neurons co-cultured with BV2 cells treated with LPS/ATP

**Fig. 6:**
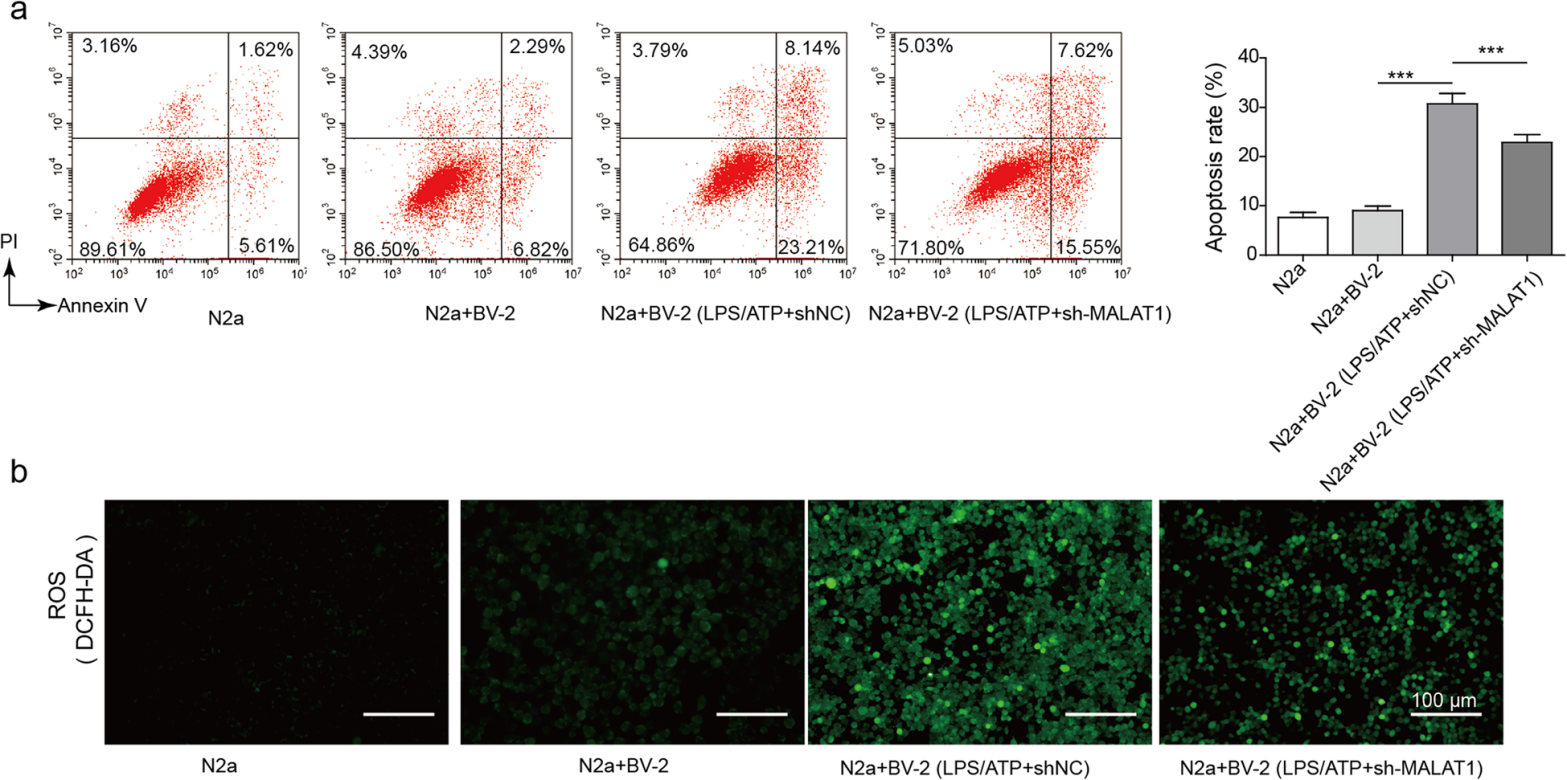
Neuroprotective effect of MALAT1 knockdown in a BV2 microglia/N2a neuro co-culture. **a** Cell apoptosis was analysed in the BV2 microglia/N2a neuro co-culture system using flow cytometry. **b** The ROS levels in N2a, N2a + BV2 cells, N2a + BV2 pretreated with LPS/ATP and shNC, and N2a + BV2 pretreated with LPS/ATP and shMALAT1. The data are presented as the means ±SD in the graph presents the relative levels from three replications.. *p < 0.05, **p < 0.01

Subsequently, we investigated the effect of MALAT1 in a BV2 microglia-N2a neuron co-culture system. LPS/ATP-pretreated BV2 microglia were transfected with shNC or sh-MALAT1 and then co-cultured with N2a neuron cells. Then, the cell apoptosis rates were analysed by flow cytometry. The results showed that LPS/ATP-pretreated BV2 cells significantly enhanced the cell apoptosis rate of N2a cells (Fig. 6a). In contrast, silencing MALAT1 in the LPS/ATP pretreated BV2 cells reversed this effect. ROS levels analysed by DCFH-DA also showed that the LPS/ATP pretreated BV2 cells significantly enhanced ROS levels in N2a cells, while knockout of MALAT1 reversed this effect (Fig. 6b). These results demonstrated that silencing MALAT1 in microglia protected neuron cells from activated microglial-induced injury.

### Silencing MALAT1 in vivo reduces the neuroinflammatory response in MPTP-challenged mice

**Fig. 7:**
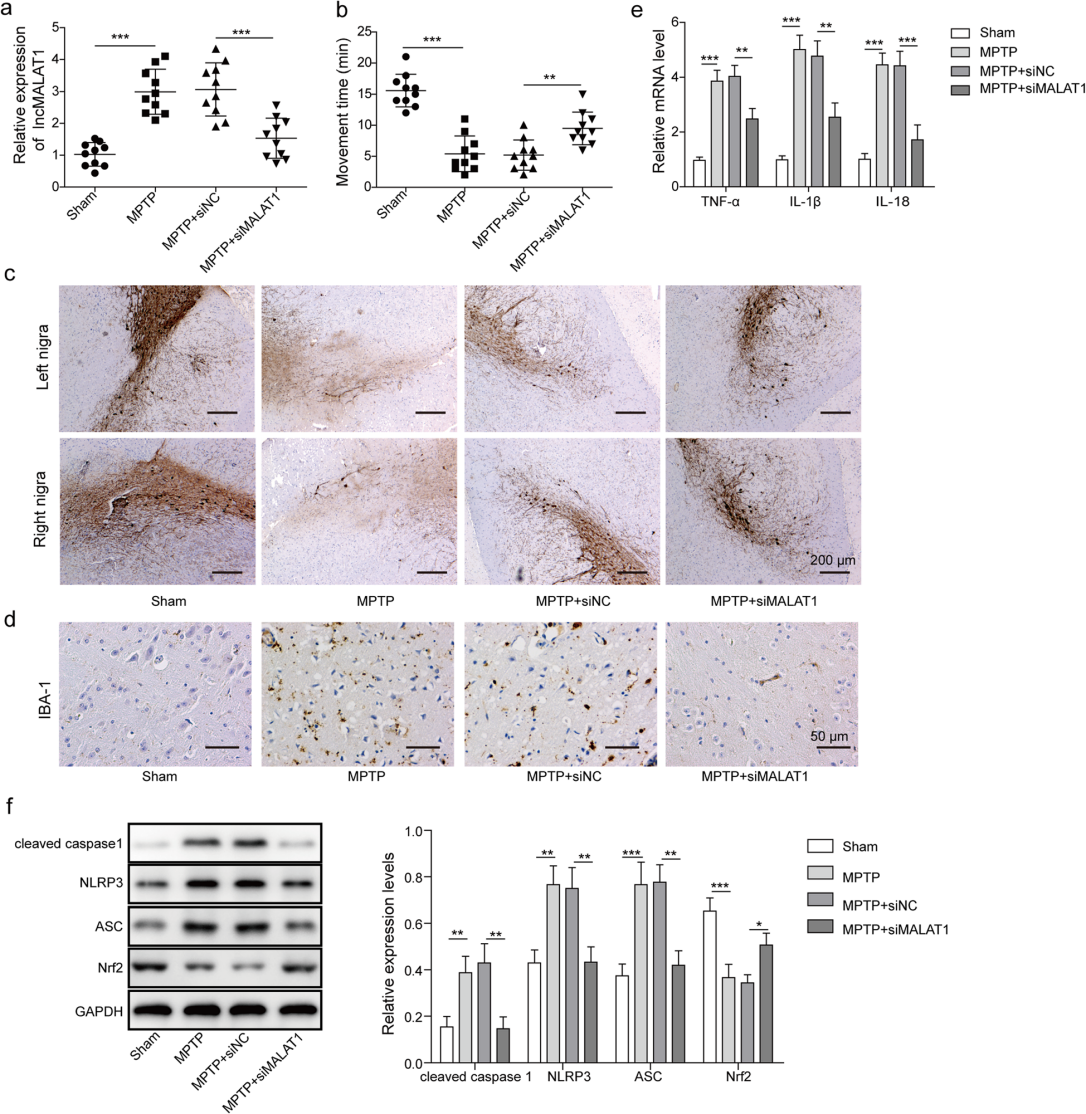
MALAT1 knockdown protects neuroinflammation in an MPTP-induced PD mouse model. After the establishment of MPTP-induced PD mouse model, siMALAT1 was injected to knockdown MALAT1 mice. siNC was used as control. **a** Expression of MALAT1 was quantified by RT-qPCR in the MPTP-induced PD mice with different treatments (**b**) Rotarod assay of the MPTP-induced PD mice with different treatments. *n* = 10 for each group. Immunohistochemical analysis of TH (**c**) and IBA-1 (**d**) in SN tissues from the MPTP-induced PD mice with different treatments. **e** Secretion of cytokines, including TNF-α, IL-1β and IL-18 were measured in MPTP-induced PD mice with different treatments by RT-qPCR. **f** Western blot analysis of the cleaved caspase 1, NLRP3, ASC and Nrf2 levels in the MPTP-induced PD mice with different treatments. The data in the graph are presented as the means ±SD as the relative levels from three replications. *p < 0.05, **p < 0.01. **a** Cell ap

Subsequently, we assessed the function of MALAT1 in MPTP-challenged mice. MALAT1 was silenced through injection with lentiviral-packaged siMALAT1, with siNC used as a control. As shown in Fig. 7a, MALAT1 expression was quantified after different treatments. The results showed that MALAT1 silencing was successful in the MPTP-induced PD mice through injection of siMALAT1. Furthermore, the rotarod test results demonstrated that silencing MALAT1 could significantly prolong the movement time in MPTP-induced PD mice compared with that observed in the control groups treated with siNC (Fig. 7b). Then, we examined the expression of TH and IBA-1 in the SN of mice with different treatments through immunohistochemical analysis. As shown in Fig. 7c&d, silencing MALAT1 in MPTP-induced PD mice significantly promoted the expression of TH and decreased that of IBA-1 in SN tissues. To evaluate the impact of MALAT1 on neuroinflammation in the MPTP-induced PD mice, the levels of pro-inflammatory cytokines, including TNF-α, IL-1β and IL-18, were analysed by RT-qPCR. The results suggested that silencing MALAT1 in the MPTP-induced PD mice significantly suppressed the secretion of these cytokines compared with that observed in the MPTP-induced PD mice treated with siNC (Fig. 7e). Through western blot analysis, the levels of inflammasome-related proteins, including cleaved caspase 1, NLRP3, ASC, and Nrf2 were measured in the SN tissues from the MPTP-induced PD mice with different treatments (Fig. 7f). We observed that the expression of cleaved caspase 1, NLRP3, and ASC was decreased when MALAT1 was silenced in the MPTP-induced PD mice. In contrast, the expression of Nrf2 was increased in the MALAT1-silenced MPTP-induced PD mice. Overall, these results suggested that silencing MALAT1 protected the neuron cells from the MPTP-induced inflammation.

## Discussion

In the present study, we demonstrated that MALAT1 was significantly and highly expressed in the MPTP-induced PD model mice as well as in BV2 cells stimulated with LPS/ATP. Moreover, the NLRP3 inflammasome was activated in the MPTP-induced PD mice and LPS/ATP-treated BV2 cells, which was coupled with decreased Nrf2 signalling. Silencing MALAT1 in the LPS/ATP-treated BV2 cells and MPTP-induced PD mice attenuated inflammasome activation. The results of a mechanistic investigation indicated that MALAT1 epigenetically inhibited Nrf2 by regulating EZH2 in the LPS/ATP-treated BV2 cells. Additionally, co-culture of N2a neuronal cells with MALAT1-silenced BV2 cells primed with LPS/ATP indicated that the neurons were protected by MALAT1 knockdown. To the best of our knowledge, this is the first time that MALAT1 has been shown to induce neuronal injury through activation of inflammasome in PD.

MALAT1 was previously reported to regulate Nrf2 to control the generation of ROS in diabetes and other ROS-induced diseases [29, 30]. For instance, Chen et al. showed that MALAT1 could significantly inhibit the expression of Nrf2 and related antioxidant genes, which promoted the generation of ROS and influenced insulin sensitivity in the treatment of diabetes. However, the regulatory mechanism remained incompletely understood. In the present study, we confirmed the effect of MALAT1 in microglial cells, which caused enhanced ROS generation through inhibition of Nrf2 expression by recruiting EZH2 to the promoter of Nrf2. Previous studies have also demonstrated that the chronic inflammation induced by activated microglial cells causes a series of neurodegenerative disorders, including PD [31]. Typically, activation of microglia is toxic to the adjacent neurons, which further promotes microglial activation and the progressive cycle of inflammation and neuron damage [32]. The cyclic interaction between microglial cells and neurons induces progressive inflammation and causes an accumulated loss of neurons over time [32]. Furthermore, there are reports showing that MALAT1 can promote activation of the inflammasome through different pathways in different disease [33–35]. For example, Li et al. discovered that MALAT1 positively regulates the expression of the NLRP3 inflammasome through modulation of miR23C in diabetic nephropathy [34]. Yu et al. noted that MALAT1 could promote NLRP3 inflammasome expression in the injured heart by sponging miR-133 [33]. Similarly, we observed that MALAT1 promoted inflammasome activation in BV2 cells through a novel pathway involving Nrf2 inhibition, which led to the induction of PD development. These results also indicated that MALAT1 could be used as a useful biomarker in the diagnosis and monitoring of PD progress, which was consistent with previous reports [36].

EZH2 is a catalytic subunit of the polycomb repressive complex 2 (PRC2) that catalyses the generation of trimethylated H3K27 (H3K27me3) from histone H3 at lysine 27 (H3K27) [37]. In addition to the transcriptional silencing effect of EZH2, the Wnt/β-catenin and Notch signalling pathways can also be enhanced by this protein [38]. Recently, MALAT1 was reported to bind EZH2 in many types of cancer, indicating the important regulatory relationship between these two elements [39–41]. For instance, MALAT1 was reported to bind EZH2 and further enhance histone 3 lysine 27 trimethylation (H3K27me3) levels at the target gene loci of EZH2 in prostate cancer cell lines [42]. EZH2 has also been reported to be involved in the regulation of inflammation. Chai et al. recently showed that EZH2 plays an important role in regulating the essential genes for inflammation in microglial activation, which induces neurodegeneration in the central nervous system [43]. In the present study, our results also validated MALAT1 binding to EZH2 in LPS-treated BV2 cells, which further recruited H3K27me3 to the gene promoter loci of Nrf2 to repress Nrf2 transcription. Although silencing MALAT1 did not alter global EZH2 expression levels, decreased binding between EZH2 and the Nrf2 promoter was observed, indicating that this is an issue worthy of further study. Many previous studies have revealed that lncRNAs regulate the function of EZH2 in a similar manner as that observe in our current study. However, the detailed mechanism associated with this activity has remained elusive. Using a series of deletion mutants of human PRC2, a previous study revealed that the basic N-terminal helix of EZH2, particularly residues 32–42 in the helix, are the most crucial for RNA binding [44]. In addition to serving as a protein scaffold, several other hypotheses have been proposed to explain how lncRNAs regulate the function of proteins, including EZH2 and RBPs. For instance, binding with lncRNA may alter the structure of protein and expose its binding site with targets [45]. Taken together, the results of the present study proved that MALAT1 can promote neuroinflammation by binding with EZH2 to epigenetically suppress Nrf2, which is followed by increased ROS generation. To the best of our knowledge, it is first report of the important role of EZH2 in regulating the expression of Nrf2 to activate microglial inflammation.

**Fig. 8:**
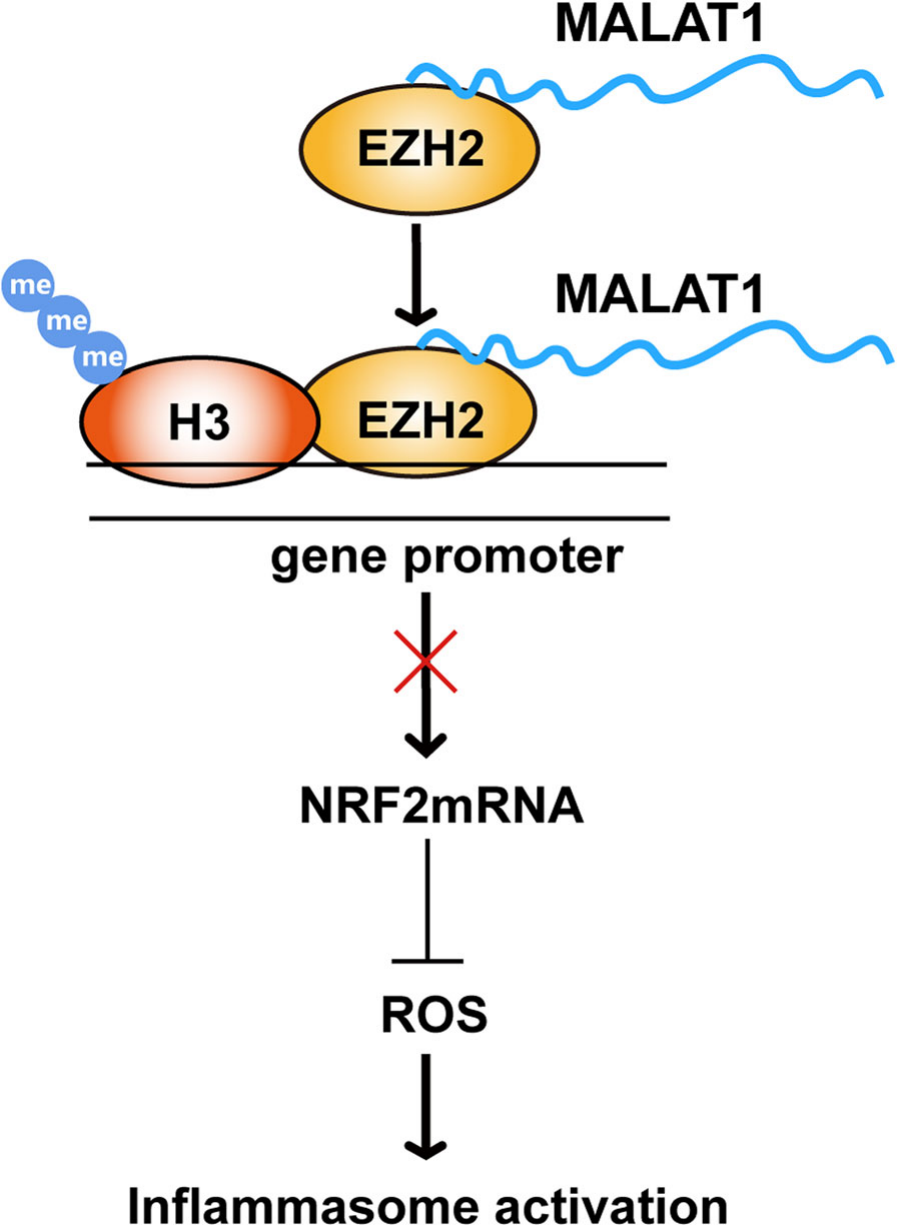
A diagram illustrating the proposed mechanisms of MALAT1 in the PD induced neuroinflammation

In summary, the results of our present study demonstrated that MALAT1 was upregulated in a PD model. MALAT1 contributed to inflammasome activation in microglial cells, triggering neuronal injury by interacting with EZH2 to regulate the Nrf2-mediated antioxidative response (Fig. 8). These findings demonstrated that the lncRNA MALAT1 may be a potential target for clinical applications against PD.

## Data Availability

All data generated or analyzed during this study are included in this published article [and its supplementary information files].

## Abbreviations

PD: Parkinson’s disease
MPTP: 1-Methyl-4-phenyl-1,2,3,6-tetrahydropyridine
MTT: 3-(4,5-Dimethylthiazol-2-yl)-2,5-diphenyltetrazolium bromide
EZH2: Enhancer of zeste homologue 2
ROS: Reactive oxygen species
LPS/ATP: Lipopolysaccharide/adenosine triphosphate
TH: Tyrosine hydroxylase
SNpc: Substantia nigra pars compacta
ncRNAs: Non-coding RNAs
lncRNAs: Long non-coding RNAs
miRNAs: MicroRNAs
MALAT1: Metastasis associated lung adenocarcinoma transcript 1
NEAT2: Nuclear-enriched abundant transcript 2
Nrf2: Nuclear factor (erythroid-derived 2)-like-2 factor
HO-1: Haem oxygenase-1
ARE: Antioxidant response element
DAB: Diaminobenzidine
DCFH-DA: Dichlorofluorescein diacetate
RT-qPCR: Quantitative reverse transcription polymerase chain reaction
RIP: RNA-binding protein immunoprecipitation
ChIP: Chromatin immunoprecipitation
